# Accessible health care for Roma: a gypsy’s tale a qualitative in-depth study of access to health care for Roma in Ghent

**DOI:** 10.1186/s12939-016-0327-7

**Published:** 2016-02-29

**Authors:** Lise G. M. Hanssens, Ignaas Devisch, Janique Lobbestael, Barbara Cottenie, Sara Willems

**Affiliations:** Department of Family Medicine and Primary Health Care, Ghent University, De Pintelaan 185, 9000 Ghent, Belgium; Integration Department, City of Ghent, Woodrow Wilsonplein 1, 9000 Ghent, Belgium

**Keywords:** Roma, Health care, Access, Trust

## Abstract

**Background:**

In general, vulnerable populations experience more problems in accessing health care. This also applies to the Roma-population. In the City of Ghent, Belgium, a relativly large group of Roma resides more or less permanently. The aim of this study is to explore the barriers this population encounters in their search for care.

**Methods:**

In this qualitative study using in-depth interviews the barriers to health care for the Roma in Ghent are explored. We interviewed 12 Roma and 13 professionals (volunteers, health care providers,…) who had regular contact with the Roma-population in Ghent. For both groups purposive sampling was used to achieve maximal variation regarding gender, age, nationality and legal status.

**Results:**

The Roma-population in Ghent encounters various barriers in their search for care. Financial constraints, not being able to reach health care and having problems to get through the complexity of the system are some of the most critical problems. Another important finding is the crucial role of trust between patient and care provider in the care-giving process.

**Conclusion:**

Roma share several barriers with other minority groups, such as: financial constraints, mobility issues and not knowing the language. However, more distinctive for this group is the lack of trust in care providers and health care in general. As a result, restraint and lack of communication form serious barriers for both patient and provider in their interaction. In order to ensure equitable access for Roma, more emphasis should be on establishing a relationship of mutual respect and understanding.

## Background

The recent admission of several central European countries to the European Union has caused an increased migration of Roma-population to West-European countries [1]. While West-European countries have different immigration rates of Roma [2], the problems they encounter in housing and supporting this population are similar.

Likewise, several reports and studies have addressed the important health inequities between Roma and the majority population. Most findings indicate that Roma have a significant shorter life expectancy, have a higher infant mortality and are more at risk for a wide variety of diseases [3–5]. This was recently confirmed in a report published by the European commission [6].

Several authors explained these health inequities by focusing on the role of the socio-economic position of the Roma-population [7, 8]. However, as Jarcuska, et al. [9] point out, worse self-rated health among Roma is also partially explained by their worse access to health care [9]. So, not only have Roma worse health than the majority population and thus a higher need for care, they also experience less access to health care facilities. This finding makes it plausible that the inequities in health experienced by Roma are, at least partially, caused by access-related problems. Identifying the barriers experienced by Roma in access to care is therefore not only important to overcome the barriers themselves but also to reduce existing health inequities.

In 2006 the European Roma Rights Centre published a report indicating that access to health care for Roma was deplorable [10]. They showed that the laws and policies that regulate access to care (in) voluntary exclude Roma from the health care benefits that are available for the majority population. Often their uncertain legal status and/or the absence of citizenship excludes them from all sorts of state subsidized social benefits, including health care.

Apart from legal regulations, Roma encounter several other barriers in their search for health care. Lack of financial resources is identified as one of the most important barriers [9]. Geographical remoteness or not having access to public transportation is another common barrier experienced by Roma [9, 10]. On top of that, the ERRC (2006) indicates that lack of information about the availability of health care is a serious issue for both Roma in their native countries as for Roma-migrants. Lastly [9], indicate that lack of trust in health care and the experience of discrimination can create an interpersonal barrier for Roma and thus worsen access. Rechel, et al. [11] have argued that lack of trust in health care can be a consequence of poor communication skills and limited cultural awareness among the health care professionals. They argue that this can cause irrational fears for the Roma-patient, since they do not understand why a certain treatment is initiated.

Other studies have tried to assess the access to care for Roma but have remained on the surface because they are based on statistical data from (population) registers or the (implicit) knowledge of experts [12]. This is not surprising as Roma are a difficult group to reach in research.

This study aims to address this gap. By using in-depth interviews with both Roma and professionals who work closely with Roma community, we want to address the following questions: what are the main barriers in access to health care for Roma in Ghent, according to Roma themselves? How are these barriers shaped by their previous experiences in their country of origin? And how do these barriers and experiences relate to the problems health care providers encounter when working with this population?

## Methods

### Design

To answer the research question unstructured in-depth interviews with both Roma and health professionals were conducted. This methodology enabled to collect data in a very flexible but focused way. The topic guide was developed within a multidisciplinary team of a GP, social worker and researchers to assure that all relevant topics were covered. We conducted a pilot interview with one of the Roma-respondents to assess the relevance of the topics included in our questionnaire. By evaluating our topic list after each interview we were able to adapt the focus of the next interview if necessary.

We constructed a separate topic list for the interviews with Roma and for health professionals, with similar topics but approaching the subject from a slightly different angle. In both groups of interviews questions were asked about the following topics: trigger(s) that led to searching health care, health beliefs, fear for illness and treatment, who do you approach when being ill, trust in health care providers, barriers when searching health care, facilitators when searching health care, discrimination and use of care in country of origin.

In order to increase the validity of the study a health mediator working for the Roma in Ghent was invited to participate in the study, was extensively trained and involved in every step of the research.

### Sampling

#### Roma

In order to reach a population as diverse as possible, purposive sampling was used. The health mediator of the City of Ghent was responsible for the recruitment of the respondents because of her professional experiences with the Roma-population and mutual relationship of trust. Based on her connections a number of persons were selected and invited face-to-face to participate in the study. Interviews were conducted until data saturation was achieved.

To make sure respondents felt at ease and to maximize participation they were interviewed at a location of choice and at a moment convenient for them.

#### Health professionals

For the sampling of the health professionals we tried to obtain a maximal variance of professionals who had experience with working with Roma. Our goal was to obtain a sample of professionals that could give insight in the different problems Roma experience when accessing care. The hypothesis was that not only medical personnel, but also social workers, and other welfare personnel could shed a different light on the same problems or/and would be confronted with different problems according to the profession they practiced. After an initial pilot-interview with the first health professional, we asked whether she knew other professionals who often worked with Roma. We repeated this question after each interview until no new names came up. We reached an extensive range of health professionals going from GPs to volunteers in charity organisations. Respondents were informed of the research by mail and afterwards invited by telephone to participate. The place and time of the interview itself was planned according to the prefrence of the respondents.

### Procedure

The Roma were interviewed by the health mediator of the City of Ghent (B.C), who previously received a training in qualitative interviewing. The health professionals were interviewed by L.H., a sociologist with training in qualitative research. Interviews took between 1 and 1,5 h.

Prior to each interview the interviewer explained the content of the project, its method and goals and repeated what was expected of the respondent. All participants were asked to sign an informed consent. For the Roma the informed consent was available in Dutch, Bulgarian and Slovak. A translator was present at those interviews where Roma-respondents had not enough knowledge of Dutch to understand or answer the questions of the interviewer.

### Coding and analysis

All interviews were audio recorded and transcribed. The first two transcripts of both groups of interviews were coded independently by B.C. and L.H. (Roma-interviews) and J.L. and L.H. (interviews with the professionals). Next, the researchers discussed the codes until consensus was achieved. This procedure was repeated after every two interviews.

The Roma-interviews were coded by the health mediator of the City of Ghent and one of the main researchers of the University department. The same researcher coded the interviews of the health professionals in cooperation with a part time-GP/part time-researcher of the University department. The data were coded through open ended coding, keeping the main goal of our research in mind. This was done using Nvivo 10 and MS Word 2010.

Analysis was undertaken by a multidisciplinary team consisting of two researchers from the department of Family Medicine and Primary Health Care, a GP and a health mediator. Trough axial coding, broader themes were identified and further analysis consisted of developing a conceptual framework based on these themes. However, to give more structure to our results, we choose to use the framework of Russell, et al. [13] to present our results.

### Ethics

This study was approved by the Ethics Committee of the University Hospital Ghent (EC registration number: B670201419905).

## Results

### Participant characteristics

Participant characteristics are described in Table 1.

**Table 1:** Characteristics of the respondents

Roma-participants	N
Sex	
Male	5
Female	7
Age	
< 35 years	5
35-50 years	6
> 50 years	2
Years in Belgium	
< 5 years	2
5-10 years	6
> 10 years	4
Residence status	
Undocumented	3
Legal	9
Family composition	
Alone	1
Lives with partner	1
Lives with partner and children	7
Lives with extended family and/or other families	3
Country of origin	
Slovakia	6
Bulgaria	4
Romania	1
Czech Republic	1
Professionals	
Sex	
Male	4
Female	9
Nature of contact with Roma	
Professional: medical	2
Professional: welfare	3
Professional: other	3
Volunteer	5

#### Roma

A total of 12 Roma were interviewed: five men and seven women. The youngest respondent was 22 years old, the oldest 66. Most of the participants lived together with a partner and/or children. Only one respondent was single and three respondents lived in a household that included more than one family. Housing conditions varied between bad, meaning homeless or living in squats, to good indicating that the respondent lived in a renting house. None of the respondents were house owner.

With regard to country of origin we included participants from the most representative nationalities of Roma in Ghent [14], which are mostly Bulgarian and Slovakian. Six respondents came from Slovakia, four from Bulgaria, one from Romania and one from the Czech Republic. The time of residence in Belgium varied from 3 to 16 years. Three respondents had a sustainable residence, meaning that their residence in Belgium was unconditional and not related to their work-status. Five of them were in possession of an E-card (identity card for European citizens residing in Belgium longer than 3 months), indicating that they must work at least five years in Belgium and they cannot receive support from social services. Three were undocumented at the time of the interviews and one was alternately undocumented or in the possession of an E-card.

#### Health professionals

Tirtheen health professionals from different disciplines were interviewed. We interviewed eight women and five men. Among them were two GPs, a health mediator, two street workers, a ‘brugfiguur’(an intermediate person, appointed by schools with a high concentration of migrants and serving as the contact person between the parents and the school), two coordinators from maternal and child care organisations and four volunteers from organisations specifically working with immigrants. They all have regular contact with Roma but the nature of that contact differed depending on the organisation in which they were active.

### Interview results

The results are structured based on the framework of access to care by Russell, et al. [13]. They proposed a conceptual model specifically designed for policy makers to evaluate primary care, and divides access into seven dimensions: availability (are sufficient PHC services available?), geography (how easily can consumers get to PHC services or services be delivered to consumers?), affordability (how easily can consumers afford PHC services?), accommodation (is the PHC organized in such a way that it suits the context from which the consumer comes?), timeliness (is the PHC service easily obtained in a timely way?), acceptability (how well does the PHC meet the sociocultural needs of consumers?) and awareness (how well do consumers understand their health issues and the PHC services available to them?).

While this framework was originally created to define access for remote communities it is also useful to apply it to communities which are not ‘remote’ in the literal sense, but which find themselves nonetheless outside mainstream society such as the Roma-population.

#### Availability

Availability as defined by Russell, et al. [13] did not come forward in the interviews and as such is not discussed here.

#### Geography

Geography as defined by Russell, et al. [13] consists of two central components: proximity and mobility. While proximity was not mentioned in the interviews, mobility did come forward in both Roma-interviews and the interviews with the professionals. Almost all Roma are entirely dependent on either public transportation or family and friends to go to the doctor. On top of that, some of the respondents indicated that they cannot always afford public transportation, limiting their means to reach a GP even further.

The professionals endorsed these results and pointed out that the problem of mobility is much more complex than ‘having the means or opportunity’ to reach a health care facility. Things such as not being able to estimate how long a bus ride will take prevents people to reach a health care facility in time often resulting in missed appointments.

*‘Waiting for the bus… Not having a good connection, who knows what can happen? I think it has a lot to do with not being able to estimate when you have to set off to be there [at a certain time].’ (professional 2)*

Some professionals indicated they try to take these matters into account, to the extent possible, by guiding people to health care facilities within walking distance of their home.

#### Affordability

It is not surprising that the financial barrier is a major issue for the Roma population. All respondents indicated that the costs of medical care tended to be very expensive to unpayable. Especially for dental care, costs were often mentioned as a reason to postpone or avoid care.

*‘Dentists who work with a third-party-payment system are lacking. Going to [the dentist] is very expensive… Very expensive. And they say you get refunded 80 % or everything for children until they are 18. But you have to have the money first to pay it.’ (Roma-respondent, male, 38 years)*

As mentioned above, not only the direct costs of care (such as consultation costs or costs of medication) but also indirect costs (such as travel costs or not having a phone or credit on the phone to make an appointment) are obstacles in getting the necessary treatment. In addition, the fee-for-service system requires that patients pay the full cost of the consultation up front, thus enhancing the fincanicial burden since the co-payment by the insurance company is only refunded after the consultation. If possible, health care workers and volunteers try to refer Roma to community health centers. In this setting, the third-party payment system is universally present, thus eliminating some of the financial constraints Roma experience.

#### Accommodation

In the interviews problems with both the organisation of health care and personal organisational aspects of the patient, clearly came forward. On the one hand, the organisation of the health care system is based on rigid rules, a strict appointment system and fragmented care for specific problems. All of these aspects are counterintuitive for this population, who often lives from day to day or problem to problem. Aside from this fact, navigating in an unknown system without the required documents or knowledge of the language often results in frustrating dead-ends.

*‘They fail to get through the reception in a hospital because… first you have to take a ticket, then at the reception you have to have the right papers or you even don’t get to see a doctor. You just don’t get through the system…’ (professional 1)*

*‘…like for example the directions in the hospital: street 1 to 20. It doesn’t say: street 1,2,3,4,… . It says: street 1 hyphen 20. I let someone try to search their way. We were looking at the directions, and he said: ‘street 15 isn’t there’. We think this is evident, but try to do this if you are not literate or have little schooling… You’re just supposed to know.’ (professional 12)*

Some of the professionals are convinced that at least part of the unnecessary use of emergency care can be explained by the complexity of the system. At emergency care there is no need for appointments and in some cases no legal documents are needed, making the intake-process considerable easier. On the other hand, the life situation of the respondents can hinder them in their search for care. For example, the fact that they don’t have someone to take care of their children if they have to go to the doctor.

#### Timeliness

The time between the need of care and the moment the patient actually gets care seems to be a crucial problem for the Roma. This problem relates closely to the matter of accommodation as described in the previous section. Because their circumstances force Roma to live from day to day, they often need or want care more quickly than the system can offer.

*‘You often have to make an appointment in advance, especially for specialists… They [Roma] only make an appointment on the moment that it is so urgent… Or on the moment that it is urgent for them and when they have to go to the appointment, which is usually two or three weeks later, there’s already another crisis. So they often forget or consciously don’t go because at that point they no longer care, there’s already something else that requires their attention.’ (professional 1)*

This contributes to the unnecessary use of emergency care, which is intensified by the fact that they often wait too long (mostly because of financial reasons) to seek regular care, which makes small problems urgent on the long term.

#### Acceptability

Acceptability attitudes or beliefs from the perspective of Roma did not explicitly come forward in the Roma interviews. Although we found no clear preferences with regard to the age, gender, ethnicity etc.… of the health care provider, one participant would not let a Moroccan GP treat him or her. Nevertheless, this does not mean that we did not find certain attitudes regarding the health care providers, only they did not relate to their personal characteristics such as described by Russell, et al. [13]. The attitudes, however, did relate to the way they were treated by the health care provider and to the extent the patient judged the health care provider to be a ‘good’ health care provider.

According to the Roma-participants, health providers are perceived as ‘acceptable’ if they can be trusted. This trust is the outcome of different aspects that relate both to the health care provider and the treatment they get and how it was given.

Most of the provider-characteristics that are valued by Roma and that hence contribute to a mutual relation of trust seem obvious. A health care provider should be empathetic, honest and treat everybody equally. Especially the latter part, equality, is very important to the respondents probably because of their experiences with discrimination in their country of origin. In general respondents say that they do not feel discriminated based on their color or ethnicity in Ghent. Even so, there are still some subtle forms of discrimination present, in particular with regard to administrative documents and insurance.

*‘I have to say… I can only speak from my own experiences, but if you go somewhere and you have a medical card [card that entitles non-legal residents without insurance to health care] and you also have another nationality, then they look like: ah, yes, that’s the one with her medical card, and then they sigh.’ (Roma-respondent, female, 22 years)*

Another element that clearly came forward in the interviews, is that respondents want their provider ‘to do their best’ for them. This seems logical, but the way they defined ‘doing their best’ stands out and is strongly related to what they expect of a treatment. A health provider who ‘does his best’ is someone who meets their expectations with regard to the treatment they think is necessary. This involves a lot of simple but concrete and technical procedures (e.g.: taking blood pressure, an echography,…). This may be the consequence of their experiences in their country of origin, where they often get sent away without treatment or solution for their problem.

Concerning the aspects of the way the treatment was given, communication is a central aspect. Communication should be present in an open and honest way so that both patient and provider are satisfied with the interaction. As already noted before, the perceived effort of the caregiver is more important than the result. Patients do not expect to understand the provider perfectly (or the provider them) as long as they feel that a certain amount of endeavor has been put in the interaction.

*‘Interviewer: What about the specialist in the hospital?*

*Respondent: With him, we don’t understand each other. He speaks very quickly, so I don’t understand anything.*

*Interviewer: What do you do if you don’t understand him?*

*Respondent: Nothing, I just look.*

*Interviewer: And your GP, does she speak more clearly? Do you understand her better?*

*Respondent: Yes, she speaks more clearly and slower. [wife adds comment]: And she speaks with her hands.’ (Roma-respondent, male, 51 years)*

One of the professionals pointed out that in the case of Roma there are additional factors that hinder fluent communication. First of all, they often do not speak Dutch, which is the first and most common language of the health care providers. Second, if there is the possibility to use an interpreter to translate the consultation, translation is only available to Bulgarian, Slovakian etc.… since none (in Ghent) or very few (in general) of the interpreters can speak Romanes. On top of that, Romanes has a very limited vocabulary and for several medical terms there is no translation possible to Romanes, meaning that patients will be confronted with words that don’t exist in their mother tongue. This creates a second language-barrier on top of their lack of knowledge of the Dutch language.

#### Awareness

Most participants know where to get the care they need. However, this is not really the consequence of ‘awareness’ as such, but more from good and close guidance of the professionals and volunteering organisations. Due to this support, most of the respondents are able to find their way in the system themselves. The fact that at some points there is little awareness becomes apparent in subtle ways. For example, not knowing various different disciplines in medicine, not knowing the name of their doctor, not being able to tell what condition they suffer from, occurs frequently.

Various respondents indicated that a lot of the information they received also came from close family or friends. While most of the time this is helpful or harmless, in some cases wrong information from their social network led to difficult situations for health care workers.

*‘Like for example: “I don’t want a medical card [card which gives right at free care] because later, if I have a job I will have to repay all the expenses.” They hear this from someone that says this and believe it…’ (professional 2)*

Awareness with regard to their health issues is also influenced by the experiences Roma had with health care in their countries of origin. Certain medical treatments, such as the use of antibiotics, have not only a scientific but also a cultural basis. This can lead to contrasting expectations when receiving care in Belgium.

*‘That’s because before, in Bulgaria they always gave antibiotics very quickly. So when D., the baby, was sick for the first time, I went there and asked for antibiotics, and they said that this was not possible, that this was not healthy and they only prescribed me drops and physiological water for the nose. I was angry at the time but then I realised that actually this was better because D. has not been sick very often since then.’ (Roma-respondent, female, 44 years)*

While awareness may be fairly poor in general within the Roma-population, professionals are convinced that it can be taught through repeated guidance. Some refer to the benefit of having school-going children since parents can learn through the information their children bring home from school. More importantly it also breaks the ongoing circle of intergenerational poverty and the absence of awareness which usually goes hand in hand with a low socio-economic status.

## Discussion

The barriers that Roma experience in access to health care seem to be twofold. On the one hand we distinguish several barriers which other vulnerable populations also witness, such as financial constraints, mobility issues and language issues. These have been documented elsewhere [15, 16] but, to a greater or lesser extent, also apply to the Roma-population. Language, for instance, actually generates a double barrier. Often Roma do not speak the country’s native language and on top of that few interpreters understand nor speak Romanes, Roma’s native language.

On the other hand some other barriers are very particular to the Roma population. Our results indicate that trust seems to be a crucial aspect. Prossibly as a consequence of lifelong discrimination (in their native countries) Roma tend to be extremely suspicious of people in general, including health care providers. The absence of trust is a problem in the sense that it is necessary to establish some level of confidence between patient and provider in order to address the questions of the patients properly.

The importance of trust for the Roma-population has occasionally been mentioned by other authors [11, 17]. In order to overcome trust-related problems in health care, measures were developed which focused on training health care workers in being more communicative and gaining a better understanding of the cultural history of the Roma [6]. This, on the one hand, would offer health care workers insight in why Roma ask particular things or behave the way they do. On the other hand, Roma would understand better why some treatments are given or not.

However, the expectation that giving more information automatically leads to a better understanding between both parties, are not always realistic. Indeed, results of a survey held among experts on Roma-related issues and –policies in 11 countries^1^, have demonstrated that the policy to facilitate access and improving health, do not always have the desirable effect [18]. For instance, policies do not automatically improve the integration of the Roma-population in public domains (such as health care), nor are policies always effective in addressing the observed problems. Although in theory some barriers to access in health care have been eliminated, in practice the barriers often remain [19]. While more and more effort has been put in improving the health status of Roma and increasing their access to health care, these results at least suggest that some policies are ineffective and do not achieve the desired results.

Instead of focusing on policies of which the effectiveness is doubtful, policymakers should consider to put more effort and resources in a method which has proven its effectiveness, i.e.: the constitution of health mediators [20]. In 1992, the health mediator program was implemented in Romania as a way to facilitate interaction between Roma and the Romanian health care providers [20, 21]. Additionally, it focused on improving the effectiveness of health interventions and preventive actions. Although gaining trust may not have been the primary goal, the health-mediator programme has been successful in ameliorating the health of Roma, by providing information and guiding patients to the health care system from a position of trust.

Mediators are mostly Roma women who are put forward by the Roma-community and have successfully completed a training program. They intermediate between the Roma-patient and the health care provider in order to facilitate interaction between both parties. Next to that, they are responsible for facilitating access to health care facilities, providing health education and implementing public health interventions.

Given its success in Romania, the mediator program has been implemented in several other countries and/or cities such as in Bulgaria, Spain, France and Brussels [6, 20].

Through this program, health mediators could succeed in what was not achieved by other policies, namely to establish a mutual relation of trust between the health care system and the Roma-population. Simultaneously they can help in resolving conflicts and clarifying miscommunications that are observed frequently. For instance, our results indicate that Roma often have extensive or unnecessary expectations and demands towards care. They only tend to regard ‘good’ treatment as treatment involving practical technical procedures (taking blood pressure, taking X-rays,…) and the prescription of drugs, in particular antibiotics. As research points out that prescription and self-use of antibiotics is generally higher in eastern-European countries compared to northern-countries [22, 23]. The latter may be the result of the prescription-habits in the country of origin. The valorisation by Roma-people of technical procedures may be a result of previous experiences but can also be a form of reassurance. Practical procedures are a tangible form of treatment and can be a handhold in a situation in which they are disadvantaged in terms of communication and knowledge. These demands and the (possible) refusal of them, result situations which create frustration for health care providers as well as for Roma. Communication, for example, is not only a barrier for Roma who are unable to explain their problems but also for caregivers who are inadequatly understood or even misunderstood, which can result in failing treatment adherence from the patient. The different expectations of patients cause similar problems. Professionals are confronted with patients asking for ‘unnecessary’ tests or medication, may perceive this as stubbornness or ‘being difficult’ since they do not know why a certain treatment or medication is asked for.

In these situations, health mediators could act as a bridge between the patient and the health care provider by translating, explaining and negotiating for both sides. In essence, the health mediator embodies a role that could also be performed by a social worker or translator, or a combination of both. The difference, and success, of a health mediator lies in the initial the position of trust the or she holds. Often other (health) care workers lack this trust. Moreover, the health mediator is also in possession of a set of skills that have been emphasized by several researchers as essential in the delivery of health care, namely profound intercultural communication skills. Some authors have suggested that limited communication skills among health care workers prevent effective health care delivery and thus contribute to health disparities [24, 25]. However, others have argued communication skills hould be supplemented by cultural competence in order to be effective. Health care workers should not only be able to educate patients about health but also be aware of the cultural perception of health and illness [26]. This includes the norms and values, gender roles and communication patterns of the minority group (in this case the Roma) that can influence their health beliefs. So health mediators have the advantage of having asufficient and adequate cultural competence and communication skills, while they also have the trust of the Roma community.

## Conclusion

One strength of this study are the in-depth interviews with Roma-respondents, who were interviewed by a trained professional which was already known to them prior the research and with whom they already established a relationship of trust. Hereby we attempted to ensure the validity of the answers given and to minimalize socially desirable answers. The in-depth interviews and qualitative nature of the study allowed us to determine the barriers Roma encounter when searching for care. This approach made it possible to explore new topics which have been neglected in previous studies (such as the difficult socio-cultural circumstances of the Roma-population in relation to health care), but also to elaborate on topics quantitative research already brought forward. Lastly, we also interviewed several health care providers which are frequently in contact with Roma people. Interviewing both Roma-respondents and professionals presented us two unique perspectives on the topic of access in health care.

However, the unstructured nature of the interviews, while also a strength, can also be a limitation to this study, since some have argued that due to the opportunities for the interviewer to intervene in the interview, there is a greater chance of bias [27]. We tried to minimalize the possibility of interviewer bias by providing an extensive training for both interviewers.

As this article shows, access to health care is still a crucial point in the health care process, especially for socially disadvantaged groups. Therefore healthcare professionals and policy makers need to continue to address access-related problems to reduce health care avoidance. In this context the importance of trust in gaining access in health care and the relationship between patient and provider can not be disregarded. Trust is necessary to establish some level of confidence between patient and provider. This also demonstrates the complex relationship between access and trust. As we indicated in the results, trust is the outcome of different aspects that relate both to the health care provider and the treatment Roma get and the way it was established. Thus, in the strict sense ‘access’, is already established. But it can also limit access in the future because negative interactions with providers can deter people from searching for care in case of prospective problems. As follows, trust is also closely related to communication, but not solely dependent on it. Communication, if satisfactory for the patient, will prevent misconception and benefit trust and once a trustworthy relationship is established, communication will facilitate communication between patient and health care provider. Nevertheless, trust (or the lack of it) is often also the result of a lifelong experience of discrimination and previous negative events. We believe that, based on the existing literature and our own research results, a combination improved communication skills and cultural competences can provide an answer to these trust issues. Communication should be more reciprocal, expectations of both the patient and health provider should be considered and stereotyping patients should be avoided. It is the task of the health care provider to initiate these things in order to obtain a more positive and open relationship with Roma-patients.”

However, the barriers which are encountered do not cease to exist when access is gained. While equitable access in all its dimensions is crucial in order to ensure that health care includes everyone in society, it is only the beginning of a much larger process of care-giving. Considering this conclusion we strongly advocate for development of more extensive health mediator programs that not only facilitate access but will benefit the whole health care seeking process for Roma patients.

## Footnotes

1 Albania, Bulgaria, Bosnia and Herzegovina, Czech Republic, Hungary, Macedonia, Montenegro, Romania, Serbia, Slovakia and Spain.
